# Kawasaki Disease Shock Syndrome in Japan and Comparison With Multisystem Inflammatory Syndrome in Children in European countries

**DOI:** 10.3389/fped.2021.625456

**Published:** 2021-03-19

**Authors:** Junko Suzuki, Kota Abe, Takuya Matsui, Takafumi Honda, Kumi Yasukawa, Jun-ichi Takanashi, Hiromichi Hamada

**Affiliations:** ^1^Department of Pediatrics, Tokyo Women's Medical University Yachiyo Medical Center, Chiba, Japan; ^2^Pediatric Critical Care Medicine, Tokyo Women's Medical University Yachiyo Medical Center, Chiba, Japan

**Keywords:** kawasaki disease, SARS-CoV-2, coronary artery, multi inflammatory syndrome-children, shock

## Abstract

Multisystem inflammatory syndrome in children (MIS-C) is a severe Kawasaki-like illness that was first linked to severe acute respiratory syndrome coronavirus 2 (SARS-CoV-2) in European countries in the spring of 2020 and has been suggested to have overlap with Kawasaki disease shock syndrome (KDSS). There are few reports of MIS-C from Asia. This observational study aimed to identify the clinical features in children presenting with KDSS in Japan over a 5-year period and to summarize similarities and differences between KDSS and MIS-C. We retrospectively collected data on patient characteristics, clinical signs and symptoms, treatment, and prognosis including coronary artery abnormalities (CAAs), which were compared with data of patients with KDSS worldwide and patients with MIS-C from a review. KDSS was identified in 6 (1.1%) of 552 patients with Kawasaki disease (KD) treated at a single institution in Japan between 2015 and 2020 (1 in 2020). In patients with KDSS in Japan or worldwide vs. patients with MIS-C, KDSS was more likely to have a diagnosis of complete KD (100, 70 vs. 6.3%), a higher incidence of CAAs (50, 65 vs. 11%), and a greater requirement for vasoactive agonists (67, 67 vs. 43%) because of circulatory shock (100, 50 vs. 26%). Both KDSS and MIS-C had good prognosis (mortality 0, 6.7 vs. 1.7%). Although KDSS in Japan and MIS-C show some overlap in clinical symptoms, they are unlikely to be the same disease entity. KDSS is more likely to have a cardiovascular phenotype with CAAs and requires treatment with cardiovascular agents.

## Introduction

The coronavirus disease 2019 (COVID-19) pandemic has had a major impact on pediatric medical care worldwide. In COVID-19, pneumonia is often mild in children, but when children develop multisystem inflammatory syndrome in children (MIS-C), also known as pediatric inflammatory multisystem syndrome temporally associated with severe acute respiratory syndrome coronavirus 2 (PIMS-TS), intensive care is required (1–3). As of spring 2020, this disease has been described in Western countries as a severe Kawasaki-like illness (4–8). Kabeerdoss et al. (9) have shown that MIS-C is a hyper-inflammatory state and can progress to MAS/cytokine storm syndrome.

Kawasaki disease is an acute vasculitis that occurs in childhood, and although its etiology is still unknown, it is known that many inflammatory cytokines are elevated. There have been many reports comparing KD and MIS-C, often stating that the two are partly similar but partly different (9–11). In particular, the overlap with KD shock syndrome (KDSS), which is KD complicated with circulatory failure and/or shock, is being discussed (12, 13). Few comparisons have been made between KDSS and MIS-C (9–13).

Currently, there are few reports on MIS-C from Asia (14, 15). The incidence of KDSS had been lower in Asia than in the US, Mexico, and European countries (16–22). There are very few case reports from Japan, which has the highest incidence of KD in the world (23). The aims of this study were to describe the clinical features and disease course in Japanese patients who presented with KDSS over a 5-year period and to discuss the similarities and differences between KDSS and MIS-C.

## Patients and Methods

This retrospective observational study was conducted at a single center in Japan. Data on patient characteristics, clinical signs and symptoms, laboratory findings, treatment, and prognosis including coronary artery abnormalities (CAAs) were collected and compared with data of patients with KDSS worldwide (19) and patients with MIS-C in a review (24).

KDSS is defined in 2009 on the basis of an age-related decrease in systolic blood pressure, a sustained decrease of 20% or more in systolic blood pressure from baseline, or clinical signs of poor blood flow (12). Our 6 cases were diagnosed based on this definition.

Patient characteristics included age, sex, and date of starting treatment. Information on major symptoms of Kawasaki disease (KD) and cardiovascular, gastrointestinal, neural, and renal symptoms were collected. The results of laboratory investigations for each patient comprised blood cell counts including neutrophils and lymphocytes, coagulation profile, inflammation-related data including the erythrocyte sedimentation rate, and ferritin, and general biochemistry including hepatic and renal function, electrolytes, and brain natriuretic peptide (BNP) or N-terminal prohormone of brain natriuretic peptide (NT-pro BNP). We also examined the Kobayashi risk score, which predicts the likelihood of the illness being refractory to intravenous immunoglobulin (IVIG) therapy (25). We also collected data on treatment and outcomes, including CAAs.

We compared the data of KDSS patients in Japan with KDSS patients worldwide (19). Then, we compared these patients with patients with MIS-C from a review (24). Laboratory data were compared between our patients and patients with MIS-C in four previous papers (8–11). The raw data available for 10 patients with MIS-C in Italy and 6 cases of KDSS in Japan were statistically analyzed using the non-parametric Wilcoxon test. Categorical data on 655 cases of MIS-C were compared with data on the 106 cases of KDSS using Fisher's exact test (19, 24).

This study was approved by the Tokyo Women's Medical University Ethics Committee (#3636).

## Results

**Figure 1:**
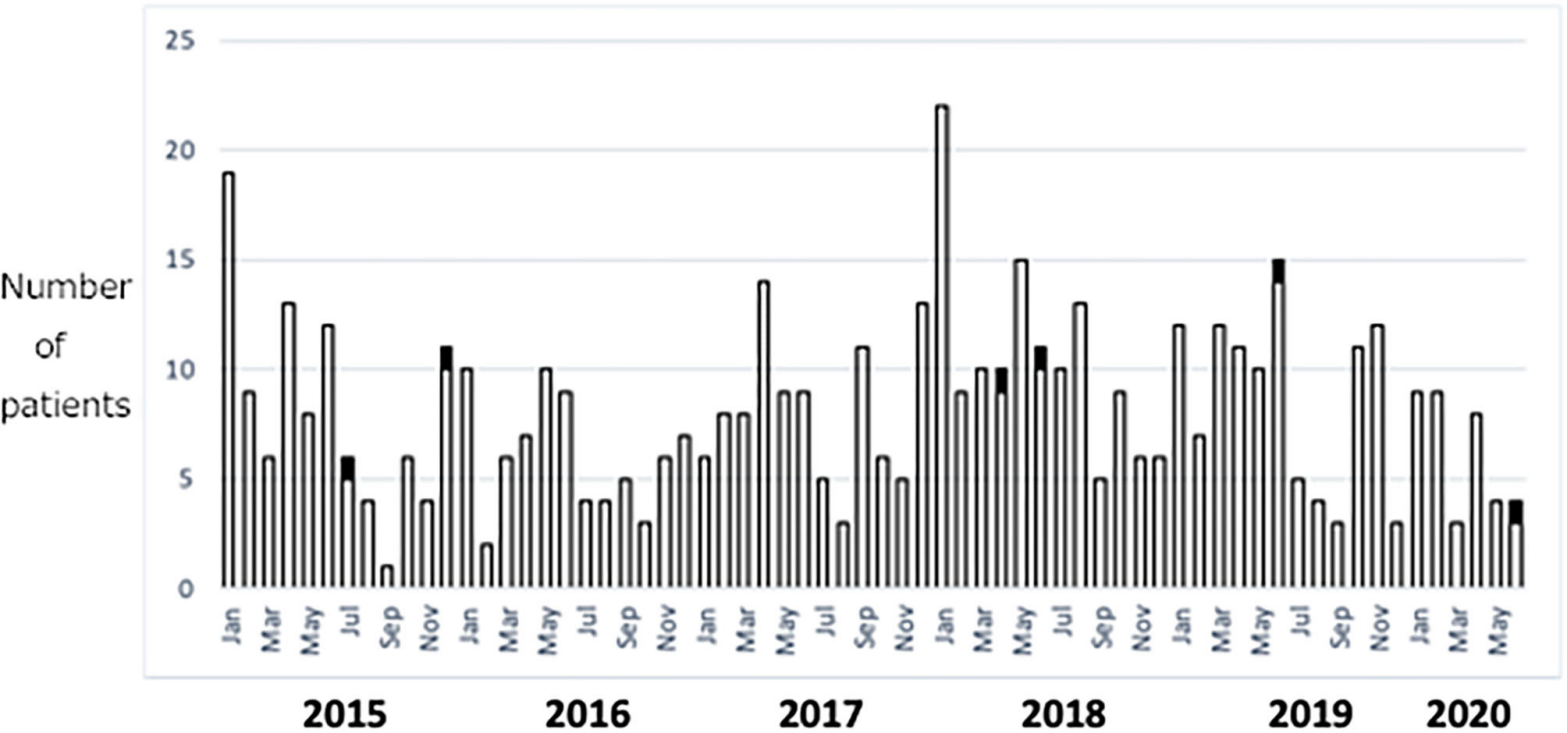
Cases of KD and KDSS between 2015 and 2020 at a single institution in Japan. White columns represent KD and black columns represent KDSS. KD, Kawasaki disease; KDSS, Kawasaki disease shock syndrome.

KDSS was identified in 6 (1.1%) of 552 patients with Kawasaki disease (KD) treated at our institution in Japan between 2015 and 2020 (2 in 2015, 2 in 2018, 1 in 2019, and 1 in June 2020). The incidence of KDSS was 1.1% or 0.09 per month (Figure 1). There was no increase in the number of patients with KD or KDSS in 2020.

### Patient Characteristics

The median age of the 6 patients with KDSS was 3.5 years in Japan, which was younger than that of 106 patients with KDSS worldwide (19). There were 655 patients with MIS-C and they were older than the KDSS patients (Table 1). The male to female ratio was 1:1 in our small cohort but 1.2:1 in the KDSS patients worldwide and MIS-C patients.

**Table 1:** Comparison between patients with KDSS and those with MIS-C.

**Type of presentation**	**KDSS in this study** ** (*n* = 6)**	**KDSS worldwide** ** (*n* = 106) (19)**	**MIS-C** ** (*n* = 655) (24)**
Age at onset	3.5 (3–12 y)	5.0 y	8 (3 mo−20 y)
Sex			
Male (%)	50%	55%	55%
Hospital stay, d	6 (4–6)	mean 7.2	4 (3–6)
Clinical Symptoms			
Fever	100%	100%	100%
Gastrointestinal	87%	75%	70%
Cardiovascular	100%	50%	51%
Neurologic	0%	54%	22%
Skin rash	100%	ND	58%
Respiratory	0%	32%	9.6%
Renal	13%	46%	ND
Met KD criteria	100%	70%	6.2%
Treatment and outcome			
Inotropes	67%	67%	45%
Invasive mechanical ventilation	0%	28%	20%
Death	0%	6.7%	1.7%

### Clinical Features

In our 6 KDSS cases, the median day of illness at the first visit was day 6 (range, 4–6). Three patients had more than five primary symptoms at the first visit and the other 3 patients had six symptoms at the start of treatment (Table 2). Diagnosis of KD was straightforward in all cases. Shock was eventually diagnosed in the 6 patients. The onset of shock was before treatment in 3 cases (Cases 4–6 in Table 2), at 5 and 8 h after the first dose of IVIG in 2 cases (Cases 1 and 3), and immediately after the start of the second dose of IVIG in 1 case (Case 2). Five (88%) of the 6 patients reported gastrointestinal symptoms, which were found to be accompanied by gallbladder enlargement on abdominal echography. One patient had acute kidney injury. The median Kobayashi risk score at admission was 6 (range, 2–9).

**Table 2:** Clinical and laboratory features of 6 patients with KDSS.

	**Case1**	**Case 2**	**Case 3**	**Case 4**	**Case 5**	**Case 6**
Date of onset	June 2015	Dec 2015	Apr 2018	June 2018	June 2019	June 2020
Age, y	12	4	6	3	3	3
Sex	M	F	M	F	F	M
Day of illness at KD diagnosis	6	4	6	6	6	5
Day of illness at shock	6^*^	9^*^	7^*^	6	6	5
Type of KD	Complete	Complete	Complete	Complete	Complete	Complete
Conjunctival injection	Yes	No	Yes	Yes	Yes	Yes
Lips and tongue	Yes	Yes	Yes	Yes	Yes	Yes
Rash	Yes	Yes	No	Yes	Yes	Yes
Swollen hands and feet	Yes	Yes	Yes	Yes	Yes	Yes
Lymphadenopathy	Yes	Yes	Yes	Yes	Yes	Yes
Other symptom						
Abdominal pain	Yes	Yes	No	Yes	No	No
Diarrhea	No	Yes	No	No	No	No
Vomiting	No	No	No	No	No	Yes
Shock	Yes	Yes	Yes	Yes	Yes	Yes
Renal failure	No	Yes	No	No	No	No
Others			A-V block			
Bacterial culture and antigen exam	GAS+	No	No	No	No	COVID-19 PCR (–)
CRP (mg/dL)	23	17.2	26.7	28.5	10.3	5.07
ESR (mm/h)	93	68	115	123	122	ND
White blood cell count	9.14	25.97	13.5	21.18	13.7	12.5
Neutrophils (%)	93	89	85	92	86	83
Lymphocytes (%)	2.7	8.9	12.1	4.6	9.1	14
Hemoglobin (g/dL)	12.6	8.6	10.2	9.2	10.9	13.4
Platelets (× 10^3^)	23.1	31.9	25.1	19.6	21.4	26.6
Albumin (g/dL)	2.7	2.0	2.0	1.7	2.8	3.5
Sodium (mEq/L)	134	128	129	135	129	136
AST (U/L)	50	19	44	33	44	39
ALT (U/L)	85	21	77	25	31	77
Total bilirubin	5.5	4.2	1.2	1.2	0.4	0.4
Ferritin (ng/mL)	713.8	548	ND	447	646	175.9
D-dimer	22.92	ND	ND	ND	ND	ND
CPK	14	235	52	11	22	36
Troponin I	0.03	ND	ND	ND	ND	15.8
BNP (pg/mL)	96.6	678	1179	1764	789.8	568
Kobayashi score	6	9	6	2	6	5
Pneumonia on chest X-ray	No	No	No	No	No	No
Ejection fraction before treatment	68	69	69	38	44	48
Mitral valve regurgitation	No	Yes	Yes	No	No	Yes
Pericardial effusion	Yes	Yes	Yes	Yes	No	No
Coronary artery lesion	Yes	Yes	Yes	No	No	No
Inotropes	Yes	Yes	Yes	Yes	No	No
Response to treatment	Yes	Yes	Yes	Yes	Yes	Yes

ALT, alanine aminotransferase; AST, aspartate aminotransferase; BNP, brain natriuretic peptide; CPK, creatine phosphokinase; CRP, C-reactive protein; ESR, erythrocyte sedimentation rate; KD, Kawasaki disease; ND, no data;

* *During/after IVIG treatment*.

In KDSS patients worldwide, 72 of 106 (70%) had complete KD. In MIS-C, the frequency of a diagnosis of complete KD was 6.3% (Table 1). The frequency of patients who met KD criteria in MIS-C was significantly lower than that in KDSS (*p* < 0.01) (24).

Gastrointestinal symptoms were common in both KDSS and MIS-C (Table 1). Cardiovascular symptoms were present in all patients with KDSS in Japan but in only about 50% of those with KDSS worldwide or MIS-C (19, 24).

### Laboratory Findings

Notable features of the KDSS were a high neutrophil ratio, a platelet count of ≤ 30 × 10^4^/μL, and a high bilirubin level (Table 2). These features have previously been reported in high-risk patients with KD (25–27).

We obtained laboratory data of patients with MIS-C from four previous papers in Europe and the US (5–8). The increase in the neutrophil ratio and the decreases in lymphocyte count and hemoglobin level were similar in KDSS and MIS-C. However, the decrease in platelets was more pronounced in MIS-C (Table 3). The difference was significant when we compared data in our 6 patients to those in 10 patients in Italy (*p* = 0.0011) (5). With regard to inflammation markers, the C-reactive protein, ferritin levels, and the erythrocyte sedimentation rate were similar in both KDSS and MIS-C. No data were reported for bilirubin in MIS-C patients. The D-dimer value as a coagulation parameter could not be evaluated due to lack of data in KDSS patients.

**Table 3:** Laboratory data.

**Type of presentation**	**KDSS in this study (*n* = 6)**	**KDSS worldwide (19) (*n* = 106)**	**MIS-C in Italy (5)** ** (*n* = 10)**	**MIS-C in UK (6)** ** (*n* = 58)**	**MIS-C in NewYork (7)** ** (*n* = 99)**	**MIS-C in US (8)** ** (*n* = 186)**
CRP (mg/dL)	20.1 (12.0, 25.8)	19.1^*^	24.1 (9.7, 27.9)	22.9 (156, 338)	21.9 (15.0, 30.0)	17.8 (12.8, 25.9)
ESR (/mm)	115 (93, 122)	61.9^*^	75.5 (51, 97)	ND	61.5 (43.0, 77.5)	65 (42, 91)
White blood cell count (× 10^3^/mm^3^)	13.6 (12.8, 19.3)	17.3^*^	10.8 (6.1)^*^	17 (12, 22)	10.4 (6.7, 14.5)	ND
Neutrophils (%)	87.5% (85.3, 91.3)	81.0^*^	84.8% (78.6, 90.3)	13 (10, 19)	82.0% (76.0–89.0)	ND
Lymphocytes (× 10^3^/mm^3^)	1.44 (1.04, 1.72)	ND	0.83 (0.46, 1.12)	0.80 (0.50, 1.50)	ND	ND
	9.0% (5.7, 11.4)				10.0% (5.0, 16.0)	
Hemoglobin (g/dL)	10.6 (9.2, 12.6)	11.1^*^	11 (1.2)^*^	9.2 (8.3, 10.3)	ND	<9.0: 48%
Platelets (× 10^4^/mm^3^)	24.1 (21.4, 26.6)	ND	13.0 (11.6, 14.2)	15.1 (10.4, 21.0)	15.5 (10.5, 23.3)	13.3 (8.8, 23.5)
ALT (IU/L)	41.5 (25, 77)	97.1^*^	56 (26, 79)	42 (26, 95)	ND	ND
Albumin (g/dL)	2.4 (2.0, 2.8)	2.4^*^	3.2 (0.3)^*^	2.4 (2.1, 2.7)	3.1 (2.5, 3.6)	2.5 (2.0, 2.9)
Ferritin (ng/mL)	548 (447, 646)	ND	893 (324, 2000)	610 (359, 1280)	ND	639 (332.7, 1178.2)
CPK (U/L)	29 (22, 52)	ND	78 (40, 87)	ND	ND	ND
Elevated BNP or NT-pro BNP (%)	100%	ND	100%	ND	90%	ND

*Median (IQR)*.

* *Mean. ND, No data*.

### Physical and Cardiovascular Function Associated With Shock

The 6 patients with KDSS had a median systolic blood pressure on admission of 78 (range, 72–93) mmHg, which is normal for children, but had minimum systolic blood pressures that were 30–39% lower than those recorded after recovery. However, the median diastolic blood pressure at admission was 41 (29–51) mmHg, which was within the normal range. The limbs were warm in all patients, indicating warm shock. On echocardiography, 3 of the 6 cases (50%) had a low cardiac ejection fraction during shock. Four patients (67%) had mild pericardial effusion. Second-degree atrioventricular block was observed in Case 3, which recovered by 1 month after onset of KD (Table 2).

Shock developed in all patients with KDSS in Japan but in 50% of patients with KDSS worldwide and in only 23% of patients with MIS-C (Table 4). BNP and NT-pro BNP levels were elevated in both KDSS and MIS-C (Table 3). Troponin data were available for only 2 patients with KDSS in Japan, precluding comparisons.

**Table 4:** Cardiovascular phenotype in KDSS and MIS-C.

**Type of presentation**	**KDSS in this study** ** (*n* = 6)**	**KDSS worldwide** (*n* = 91) (19)^*^	**MIS-C** ** (*n* = 655) (24)**
Ejection fraction * ≤* 55	50%	45%^**^	32%
Myocarditis	100%	ND	23%
Mitral valvular regurgitation	50%	14%	ND
Coronary artery abnormalities	50%	65%	11%^†^
Circulatory shock	100%	50%	26%

* *Patients in whom echocardiography was obtained*.

** *Ejection fraction <50%*.

† *Among 482 patients in whom echocardiography was obtained. ND, No data*.

### Treatment

In the 6 patients with KDSS, treatment was started on median day 6 of illness. All patients received IVIG plus aspirin. Cyclosporine A was administered with IVIG and aspirin in Case 6 (Table 2). Three of the 6 patients were resistant to the initial dose of IVIG and required up to third-line treatment for KD. Four of the 6 patients received inotropic agents, including dobutamine and a phosphodiesterase (PDE) inhibitor. The response to these agents was very good in all cases. The first patient received dobutamine 3 μg/kg/h, which was tapered over 4 days (Case 1 in Table 2). The second patient received a combination of dobutamine and a PDE inhibitor (Case 2); the initial dose of dobutamine was 3γ and that of the PDE inhibitor was 0.1γ, with a treatment duration of 4 days. The third patient received dobutamine over a period of 26 h (Case 3). A combination of dobutamine and a PDE inhibitor was administered in the fourth patient; the respective initial doses were 2 and 0.05γ and the treatment duration was 4 days (Case 4). No inotropic agent was used in Case 5 and Case 6. Systolic blood pressure during use of inotropic agents was over 110 mmHg in 3 of the 4 patients who received inotropic support.

In the KDSS patients worldwide, 67% were treated with inotropic agents. In the MIS-C patients, 45% were treated with inotropic agents, which is a smaller proportion than in the patients with KDSS in Japan (Table 1).

### Coronary Artery Abnormalities and Mortality

Three of the 6 patients with KDSS in Japan (50%) had CAAs. Case 1 had a giant aneurysm, and Cases 2 and 3 had transient dilation. This high incidence of CAAs was similar in KDSS patients worldwide (Table 4) (19). Patients with MIS-C had CAAs at a frequency of 11% among 482 patients in whom echocardiography was obtained (24).

There were no deaths from KDSS in Japan, whereas 6.7% of patients with KDSS worldwide and 1.7% of patients with MIS-C died. The US Centers for Disease Control and Prevention reported 20 deaths among 1,027 cases of MIS-C (2%) as of October 12, 2020 (28).

## Discussion

There are few reports of KDSS in Japan (23), and there is insufficient information on the differences in clinical symptoms between cases in Japan and cases in Europe and the US. We have encountered 6 cases in 5 years based on the definition of Kanegaye et al. (12). The frequency in Japan is 1.1%, which is less than the rate of 5–7% reported in Europe and the US (16–18). The frequency is also 1–3% in Taiwan and China, and there are reports suggesting that KDSS is less common in Asians (20–23). The features of KDSS identified in this study, such as patients being older than those with KD without shock, presence of more abdominal symptoms, meeting the diagnostic criteria for KD, likelihood of being refractory to IVIG, and use of vasoactive agents in two-thirds of cases, are consistent with the features reported in a review of KDSS centered on Europe and the US (19). However, in the review, the frequency of requirement for mechanical ventilation (28%) and the mortality rate (6.8%; 7 patients, 3 of whom had myocardial infarction) were different from our experience. Access to medical care is good in Japan, and diagnosis of KD is rapid given the relatively high frequency of the disease in Japan. On average, treatment is started on day 4 of illness. Early treatment may help to reduce mortality.

KD, and especially KDSS, has received considerable attention due to the outbreak of MIS-C in 2020. Two to 4 weeks after the onset of SARS-CoV-2 infection, inflammation spreads to multiple organs. Many children present with disorders affecting the circulatory, gastrointestinal, renal, and other organ systems, and this condition is diagnosed as MIS-C or PIMS-TS (1–3). MIS-C was reported to have clinical symptoms similar to those of KDSS (4–9). Many more cases of KDSS have been registered since March 2020 than were diagnosed until 2019 (4–8). However, there are very few reports of MIS-C in Asia, including Wuhan, which is the birthplace of SARS-CoV-2 (14, 15). As shown here, the number of cases of KDSS did not increase in 2020 in Japan. In 2020, we had 1 case of KDSS, which was negative for SARS-CoV-2 by polymerase chain reaction (antibodies untested). The total number of KD cases in Japan is lower in 2020 than in 2019.

Patients with KDSS are older than those with KD without shock, but the percentage under 5 years of age was reported to be 56% in the review of KDSS (19) and was 67% in our case series. In contrast, about 25% of patients with MIS-C were over 12 years old. It has been reported that 55% of patients with KDSS worldwide are boys (19) and that the sex ratio for MIS-C is the same (24).

The pathophysiology of KDSS is understood to be mainly arteritis and myocarditis with lesions in multiple organs. In our experience, the limbs are warm and KDSS is considered to be a mixed type of shock due to decreased cardiac contraction and hyperpermeability of the peripheral arteries (18). In MIS-C, whether or not the pathophysiology of shock is similar to that of KDSS is an important question.

The rate of CAAs is much higher in KDSS than in MIS-C. The frequency in KDSS was significantly higher than that in the MIS-C (*p* < 0.01) (19, 24). Some patients with MIS-C present with coronary aneurysm similar to KD; however, the definition of MIS-C differs slightly between the US, Europe, and the World Health Organization, and since it is not strict, some KD cases could be mixed in with MIS-C cases. SARS-CoV-2 infection promotes release of an angiotensin-converting enzyme receptor in the vascular endothelium and has been reported to cause vasculitis (29), but we would like to know whether the main focus of inflammation is arteries or veins. Recently, Consigio et al. (30) published a comprehensive analysis of peripheral blood immune cells and blood cytokines in patients with KD and MIS-C and reported that the inflammatory response in MIS-C differs from that in KD with respect to T cell subsets, some cytokines, and biomarkers associated with arterial damage.

Gastrointestinal symptoms are also common in KDSS, particularly vomiting and abdominal pain, which are caused by cholestasis (19). We believe these symptoms may also be caused by gastrointestinal hypoperfusion due to heart failure. In MIS-C, the main symptom is reported to be diarrhea (4–8). We would like to know about gallbladder swelling and the blood bilirubin level in MIS-C. Renal failure, nervous system disorders, and respiratory symptoms were reported to occur with similar frequency in KDSS and the MIS-C (5–8, 19, 24).

In our study, the ferritin level, which is important for the diagnosis of MIS-C, was not markedly different between patients with MIS-C and those with KDSS. However, serum ferritin levels were extremely high in some patients with MIS-C, and we consider that further investigation is needed before we can draw a conclusion. Platelet counts were often below 300,000/μL in both diseases but were lower in patients with MIS-C. Although measurements of D-dimer and fibrinogen were insufficient in this study, we think that the pathophysiology of MIS-C is closer to that of macrophage-activating syndrome.

Both diseases had much in common in terms of treatment, with management in the intensive care unit in 70–80% of cases and ventilation management in 20–30%; about 50% were refractory to IVIG, and steroids were used in 35%. Use of vasoactive agents was significantly higher in the KDSS patients than in the MIS-C patients (67 vs. 43%; Table 1), which reflects the fact that the phenotype of KDSS includes more severe heart failure. The mortality rate for the two diseases is similar at 0–6.7% for KDSS and 0–2% for MIS-C (4–8, 28) (Table 1).

This study has several limitations. First, the number of KDSS cases was small, and some of our findings were different from those reported previously for KDSS, so we did not adequately perform statistical analysis. Therefore, we cannot reach any definitive conclusion based on our results. Second, the diagnostic criteria for both KDSS and MIS-C are symptom-based diagnoses, and their clinical symptoms overlap. Of course, there may be patients who meet both diagnostic criteria for both diseases. Third, the study had a retrospective design, which meant that only a limited number of features could be compared between KDSS and MIS-C.

## Conclusion

KDSS in Japan and MIS-C in European countries have some similar clinical signs and symptoms, but despite this overlap are unlikely to be the same disease entity, in light of the diagnostic criteria for KD and the incidence of CAAs, which are features of the most important phenotype of KD. KDSS has a predominantly cardiovascular phenotype and requires more treatment with circulatory agents. Analysis of the pathophysiology of MIS-C has started, and further studies focusing on vasculitis are awaited to elucidate the size of blood vessels and whether arteries or veins are mainly involved.

## Data Availability Statement

The original contributions presented in the study are included in the article/supplementary material, further inquiries can be directed to the corresponding author/s.

## Ethics Statement

The studies involving human participants were reviewed and approved by Tokyo Women's Medical University Ethical Committee. Written informed consent from the participants' legal guardian/next of kin was not required to participate in this study in accordance with the national legislation and the institutional requirements.

## Author Contributions

JS analyzed the data and wrote the manuscript. KA analyzed the data. TM, KY, and TH critically reviewed the manuscript. J-iT reviewed the research and monitored ethical issues. HH designed the study and revised the manuscript. All authors contributed to the article and approved the submitted version.

## Conflict of Interest

The authors declare that the research was conducted in the absence of any commercial or financial relationships that could be construed as a potential conflict of interest.

## References

[1] European Centre for Disease Prevention and Control. Rapid Risk Assessment: Paediatric Inflammatory Multisystem Syndrome and SARS-CoV-2 Infection in Children. (2020). Available online at: https://www.ecdc.europa.eu/en/publications-data/paediatric-inflammatory-multisystem-syndrome-and-sars-cov-2-rapid-risk-assessment (accessed October 17, 2020).

[2] Royal College of Paediatrics and Child Health. Guidance: Paediatric Multisystem Inflammatory Syndrome Temporally Associated With COVID-19. Available online at: https://www.rcpch.ac.uk/resources/guidance-paediatric-multisystem-inflammatory-syndrome-temporally-associated-covid-19-pims (accessed October 17, 2020).

[3] World Health Organization. Multisystem Inflammatory Syndrome in Children and Adolescents With COVID-19. (2020). Available online at: https://www.who.int/news-room/commentaries/detail/multisystem-inflammatory-syndrome-in-children-and-adolescents-with-covid-19 (accessed October 17, 2020).

[4] Riphagen S Gomez X Gonzalez-Martinez C Wilkinson N Theocharis P. Hyoerinnflammatory shock in children during COVID-19 pandemic. Lancet. (2020) 395:1607–8. 10.1016/S0140-6736(20)31094-1 32386565 PMC7204765

[5] Verdoni L Mazza A Gervasoni A Martelli L Ruggeri M Ciuffreda M. An outbreak of severe Kawasaki-like disease at the Italian epicentre of the SARS-CoV-2 epidemic: an observational cohort study. Lancet. (2020) 395:1771–8. 10.1016/S0140-6736(20)31103-X 32410760 PMC7220177

[6] Whittacker E Bamford A Kenny J Kaforou M Jhones CE Shah P. Clinical characteristics of 58 children with a pediatric inflammatory multisystem syndrome temporally associated with SARS-CoV-2. JAMA. (2020) 324:259–69. 10.1001/jama.2020.10369 32511692 PMC7281356

[7] Dufort EM Koumans EH Chow EJ Rosenthal EM Muse A Rowlands J. Multisystem inflammatory syndrome in children in New York State. N Engl J Med. (2020) 383:347–58. 10.1056/NEJMoa2021756 32598830 PMC7346766

[8] Feldstein LR Rose EB Horwitz SM Collins JP Newhams MM Son MBF. Multisystem inflammatory syndrome in U.S. children and adolescents. N Engl J Med. (2020) 383:334–46. 10.1056/NEJMoa2021680 32598831 PMC7346765

[9] Kabeerdoss J Pilania RK Karkhele R Sathish Kumar T Danda D Singh S. Severe COVID-19, multisystem inflammatory syndrome in children, and Kawasaki disease: immunological mechanisms, clinical manifestations, and management. Rheumatol Int. (2021) 41:19–32. 10.1007/s00296-020-04749-4 33219837 PMC7680080

[10] Haslak F Yildiz M Adrovic A Sahin S Barut K Kasapçopur Ö. A recently explored aspect of the iceberg named COVID-19: multisystem inflammatory syndrome in children (MIS-C). Turk Arch Pediatr. (2021) 56:3–9. 10.5152/TurkArchPediatr.2020.20245 PMC8114613 34013222

[11] Schvartz A Belot A Kone-paut I. Pediatric inflammatory multisystem syndrome and rheumatic diseases during SARS-CoV-2 pandemic. Front Pediatr. (2020) 8:605807. 10.3389/fped.2020.605807 33344389 PMC7746854

[12] Kanegaye JT Wilder MS Molkara D Frazer JR Pancheri J Tremoulet AH. Recognition of a Kawasaki disease shock syndrome. Pediatrics. (2009) 123:e783–9. 10.1542/peds.2008-1871 19403470 PMC2848476

[13] Dominguez SR Friedman K Seewald R Anderson MS Willis L Glodé MP. Kawasaki disease in a pediat-ric intensive care unit: a case-control study. Pediatrics. (2008) 122:e786–90. 10.1542/peds.2008-1275 18809597

[14] Rauf A Vijayan A John ST Krishnan R Latheef A. Multisystem inflammatory syndrome with features atypical Kawasaki dieseaseduring COVID-19 pandemic. Indian J Peditr. (2020) 87:745–7. 10.1007/s12098-020-03357-1 32462354 PMC8823324

[15] Kim YJ Park H Choi YY Kim YK Yoon Y Kim KR. Defining association between COVID-19 and the multisystem inflammatory syndrome in children through the pandemic. J Korean Med Sci. (2020) 35:e204. 10.3346/jkms.2020.35.e204 32508068 PMC7279946

[16] Gatterre P Oualha M Dupic L Iserin F Bodemer C Lesage F. Kawasaki disease: an unexpected etiology of shock and multiple organ dysfunction syndrome. Intensive Care Med. (2012) 38:872–8. 10.1007/s00134-012-2473-8 22273753

[17] Lin MT Fu CM Huang SK Huang SC Wu MH. Population-based study of Kawasaki disease shock syndrome in Taiwan. Pediatr Infect Dis J. (2013) 32:1384–6. 10.1097/INF.0b013e31829efae6 23748909

[18] Gámez-González LB Murata C Muñoz-Ramírez M Yamazaki-Nakashimada M. Clinical manifestations associated with Kawasaki disease shock syndrome in Mexican children. Eur J Pediatr. (2012) 172:337–42. 10.1007/s00431-012-1879-1 23152158

[19] Gamez-Gonzalez LB Moribe-Quintero I Cisneros-Castolo M Varela-Ortiz J Muñoz-Ramírez M Garrido-García M. Kawasaki disease shock syndrome: unique and severe subtype of Kawasaki disease. Pediatr Int. (2018) 60:781–90. 10.1111/ped.13614 29888440

[20] Chen PS Chi H Huang FY Peng CC Chen MR Chiu NC. Clinical manifestations of Kawasaki disease shock syndrome: a case-control study. J Microbiol Immunol Infect. (2015) 48:43–50 10.1016/j.jmii.2013.06.005 23927822

[21] Zhang MM Shi L Li XH Lin Y Liu Y. Clinical analysis of Kawasaki disease shock syndrome. Chin Med J. (2017) 130:2891–2. 10.4103/0366-6999.219151 29176153 PMC5717875

[22] Ma L Zhang YY Yu HG. Clinical manifestations of kawasaki disease shock syndrome. Clin Pediatr. (2018) 57:428–35. 10.1177/0009922817729483 28905639

[23] Ono R Shimizu M Yamamoto K Umehara N Manabe A. Kawasaki disease shock syndrome: case report and cytokine profiling. Pediatr Int. (2019) 61:620–2. 10.1111/ped.13864 31216599

[24] Kaushik A Gupta S Sood M Sharma S Verma S. A systematic review of multisystem inflammatory syndrome in children associated with SARS-CoV-2 infection. Pediatr Infect Dis J. (2020) 39:e340–6. 10.1097/INF.0000000000002888 32925547

[25] Kobayashi T Inoue Y Takeuchi K Okada Y Tamura K Tomomasa T. Prediction of intravenous immunoglobulin unresponsiveness in patients with Kawasaki disease. Circulation. (2006) 113:2606–12. 10.1161/CIRCULATIONAHA.105.592865 16735679

[26] Egami K Muta H Ishii M Suda K Sugahara Y Iemura M. Prediction of resistance to intravenous immunoglobulin treatment in patients with Kawasaki disease. J Pediatr. (2006) 149:237–40. 10.1016/j.jpeds.2006.03.050 16887442

[27] Sano T Kurotobi S Matsuzaki K Yamamoto T Maki I Miki K. Prediction of non-responsive to standard high-dose gamma-globulin therapy in patients with acute Kawasaki disease before starting initial treatment. Eur J Pediatr. (2007) 166:131–7. 10.1007/s00431-006-0223-z 16896641

[28] Health Department – Reported Cases of Multisystem Inflammatory Syndrome in Children (MIS-C) in the United States. Available online at: https://www.cdc.gov/mis-c/cases/index.html (accessed March 8, 2021).

[29] Wadman M Couzin-Frankel J Kaiser J Matacic C. A rampage through the body. Science. (2020) 368:356–60. 10.1126/science.368.6489.356 32327580

[30] Consiglio CR Cortugno N Sardh F Pou C Amodio D Rodriguez L. The immunology of multisystem inflammatory syndrome in children with COVID-19. Cell. (2020) 183:1–14. 10.1016/j.cell.2020.09.016 32966765 PMC7474869

